# Evolved role of the cardiovascular intensive care unit (CICU)

**DOI:** 10.1186/s40560-017-0271-7

**Published:** 2017-12-22

**Authors:** Shunji Kasaoka

**Affiliations:** Department of Emergency and General Medicine, Kumamoto University Hospital, 1-1-1 Honjo, Chuo-ku, Kumamoto, 860-8556 Japan

**Keywords:** Acute myocardial infarction, Cardiovascular intensive care, Cardiovascular disease, Coronary care unit, Intensive care unit

## Abstract

Cardiovascular intensive care refers to special systemic management for the patients with severe cardiovascular disease (CVD), which consists of heart disease and vascular disease. CVD is one of the leading causes of death in the world. In order to prevent death due to CVDs, an intensive care unit for severe CVD patients, so-called cardiovascular intensive care unit (CICU), has been developed in many general hospitals. The technological developments of clinical cardiology, such as invasive hemodynamic monitoring and intracoronary interventional procedures and devices, have resulted in evolution of intensive care for CVDs. Subsequently, severe CVD patients admitted to CICU are increasing year by year. Dedicated medical staff is required for CICU in order to perform best patient management. It is necessary for optimal patient care to select effective means from various hemodynamic tools and to adjust the usage according to the clinical situation such as cardiogenic shock and acute heart failure. Furthermore, the patients in the CICU often have various complications such as respiratory failure and renal failure. Therefore, medical staffs who work at CICU are required to have the ability to practice systemic intensive care.

## Background

Cardiovascular intensive care refers to special systemic management for the patients with severe cardiovascular disease (CVD), which consists of heart disease and vascular disease. The heart diseases include coronary artery diseases (CAD) such as angina and myocardial infarction, cardiomyopathy, myocarditis, heart arrhythmia, hypertensive heart disease, and valvular heart disease. The vascular diseases include aortic dissection, aortic aneurysm, peripheral artery disease, etc.

It is reported that CVD is the second leading cause of mortality worldwide, accounting for 17 million deaths in 2013 [1]. Although the risk factors for the development of CVD are similar throughout the world, improvement of cardiovascular risk factors such as smoking and obesity is effective to reduce the incidence of CVD.

In recent years, in order to prevent death due to CVDs, an intensive care unit for severe CVD patients, so-called cardiovascular intensive care unit (CICU), has been developed in many general hospitals [2]. In this article, I will review the history of the CICU and discuss the recent changes in cardiovascular intensive care.

## Epidemiology of cardiovascular diseases

CVDs consist of various heart diseases and vascular diseases. The pathogenesis of onset depends on each CVD. There are many risk factors for heart diseases: age, smoking, obesity, hypertension, diabetes mellitus, and hyperlipidemia. These risk factors increased from 12.3 million deaths (25.8%) in 1990 to 17.9 million deaths (32.1%) in 2015 [3]. Many important cardiovascular risk factors are modifiable by lifestyle change and drug treatment such as prevention of hypertension, hyperlipidemia, and diabetes. It is estimated that 90% of CVD is preventable [4].

CVDs are common among elderly people. In the USA, it is reported that 11% of people between 20 and 40 years old have CVD, while 37% between 40 and 60 years, 71% between 60 and 80 years, and 85% over 80 years have CVD [5].

According to vital statistics of the Ministry of Health, Labor and Welfare in Japan, CVD is the second leading cause of death in Japan. About 200,000 people died due to CVDs in 2015. In addition, approximately 60,000 Japanese people have an out-of-hospital cardiac arrest due to CVDs every year and the overall life-saving rate is still low [6].

Since the CVDs contain many fatal emergency diseases, coronary care unit (CCU) was established as a facility responsible for intensive care in the acute phase in order to improve the outcome of the CVDs.

## Progress from the coronary care unit to the cardiovascular intensive care unit

The development of CCU in the mid-twentieth century was a major advance in cardiology practice [7]. CCU was developed in the 1960s when it became clear that close monitoring by specially trained staff, cardiopulmonary resuscitation (CPR), and medical interventions can reduce mortality due to CVD complications such as cardiogenic shock and fatal arrhythmias.

CCU, which was initially established as a separate unit for the early detection and treatment of arrhythmias complicating AMI, currently provides the setting for the monitoring and treatment of a wide variety of critical CVD states. Therefore, the CCU has come to be called the CICU. The role of cardiovascular intensive care has evolved with the rapid progress of diagnostic and therapeutic strategies in the practice of clinical cardiology [7]. The technological developments of clinical cardiology, such as invasive hemodynamic monitoring and intracoronary interventional procedures and devices, have resulted in evolution of intensive care for CVDs. Subsequently, severe CVD patients admitted to CICU are increasing year by year.

**Fig. 1:**
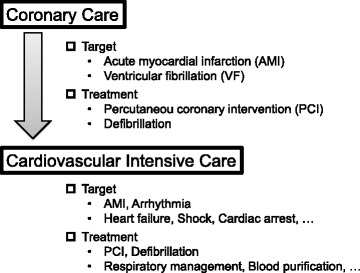
Evolved role of the cardiovascular intensive care

Figure 1 displays my concept about cardiovascular intensive care. In the era of CCU, the main target patients were acute myocardial infarction (AMI). Percutaneous coronary intervention (PCI) and defibrillation were important treatments. Subsequently, as the target patients spread to heart failure, shock, out-of-hospital cardiac arrest, etc., the need for cardiovascular intensive care including respiratory management and blood purification therapy increased.

## Features of cardiovascular intensive care

The CICU is a hospital ward specialized in the care of patients with severe heart diseases, such as AMI, cardiomyopathy, and arrhythmias. Those patients often complain of heart failure and cardiogenic shock. Therefore, the severe CVD patients need continuous monitoring and intensive care.

The main feature of CICU is the availability of the continuous monitoring of the cardiac rhythm by electrocardiography (ECG). This allows early intervention with medication, cardioversion, or defibrillation, improving the prognosis of the severe CVD patients. Furthermore, cardiovascular intensive care needs to have various kinds of diagnostic medical equipment as shown in Table 1. Also, therapeutic equipment necessary for cardiovascular intensive care is shown in Table 2. In addition to circulation management, systemic management is required in the CICU. So, it is necessary to prepare a ventilator and a blood purification device as well as the auxiliary circulation devices, such as intra-aortic balloon pump (IABP) and percutaneous cardiopulmonary support system (PCPS) in the CICU. Recently, it is also indispensable to provide the equipment for performing targeted temperature management for the patients resuscitated from cardiogenic out-of-hospital cardiac arrest (OHCA) [8].

**Table 1:** Diagnostic equipment necessary for cardiovascular intensive care

• Bedside monitoring system: ECG, blood pressure, respiratory rate	
• Pulse oximeter (SpO_2_)	
• Thermometer	
• Twelve-lead electrocardiography	
• Cardiac ultrasound device	
• Cardiac output measurement system: Swan-Ganz catheter	
• Portable X-ray imaging equipment	
• Doppler blood flow meter	
• Blood gas analyzer

**Table 2:** Therapeutic equipment necessary for cardiovascular intensive care

• Defibrillator	
• Cardiac pacemaker	
• Noninvasive ventilation system	
• Mechanical ventilator	
• Blood purification device	
• Intra-aortic balloon pump	
• Percutaneous cardiopulmonary assist device	
• Temperature management system	

A dedicated medical staff is required for cardiovascular intensive care in order to perform best patient management. In Japan, cardiologists certified by the Japan Circulation Society are assigned to CICU. In addition, nurses and technicians who are trained on professional care of CVD patients are also assigned. In order to provide the best patient management, team medical care through cooperation of medical staff in the CICU is indispensable. The CICU physician staff needs the ability to evaluate electrocardiograms and cardiac functions by echocardiography.

In recent years, cardiologists have been required not only circulation management but also systemic intensive care practices such as respiratory management and infusion management. Cooperation between cardiologists and intensive care specialists is also important to cope with CVD patients with various complications, such as respiratory failure, renal failure, and sepsis. I believe that systemized training related to general intensive care is necessary so that CICU staff can master the use of diagnostic and therapeutic medical equipments shown in Tables 1 and 2.

In the USA, coronary care units are usually subsets of intensive care units (ICU) dedicated to the care of critically ill cardiac patients**.** These units are usually present in hospitals that routinely engage in cardiothoracic surgery. It is reported that noncardiovascular disease-related acuity has significantly increased in the CICU and may be influencing mortality [9].

Recently, it is reported that lessons learned from advances in cardiovascular intensive care can be broadly applied to address the urgent need to improve outcomes and efficiency in a variety of health care settings [10]. The CICU is a high-risk environment that admits complex patients suffering from acute conditions that can become life-threatening at any moment. It is reported that simulation-based teaching program yields many benefits for cardiac intensive care units, allowing professionals to acquire not only procedural skills specific to the practice but also confidence and competence as members of an efficient and skilled resuscitation team [11].

## Monitoring of the heart and vascular system

The most important monitor in cardiovascular intensive care is an electrocardiogram that evaluates the cardiac rhythm of the CVD patients. In addition, hemodynamic monitoring of invasive arterial pressure and pulmonary artery pressure may be required in the CVD patients complicated with cardiogenic shock or acute heart failure. Hemodynamic evaluation is an important factor in the severity assessment of those patients. For CICU medical staff, it is necessary for optimal patient care to select effective means from various hemodynamic tools and to adjust the usage according to the clinical situation [12].

Since the introduction in the 1970s, pulmonary artery catheter has been commonly utilized for hemodynamic monitoring in the critically ill patient, especially in the adult population [13]. The standard pulmonary artery catheter, as developed by Drs. Swan and Ganz, has four lumens along its length, and these lumens allow for the assessment of hemodynamic data in various places along the right-sided circulation [14]. Data available include right atrium pressure, right ventricle pressure, pulmonary artery pressure, and pulmonary capillary wedge pressure. Using these variables and measured values of heart rate, systemic arterial pressure, and cardiac output, numerous hemodynamic variables can be calculated, including pulmonary and systemic vascular resistance. Cardiac output is most commonly measured with the pulmonary artery catheter using the thermodilution technique. Advantages of the thermodilution method include its validated reliability and its ease of use at the bedside of CCU.

Furthermore, cardiac output can be measured with new technology, which is estimated by analysis of the pulse contour from an arterial waveform, since the systolic portion of the waveform reflects stroke volume (SV) [12]. In recent years, these devices have been used for hemodynamic monitoring in the CICU.

Although the use of invasive hemodynamic monitoring has declined in recent years, it is possible to obtain useful information for assessing the pathology and severity of the CVDs and determining the treatment policy for the critically ill patients.

## Targeted temperature management for the patients with out-of-hospital cardiac arrest in the CICU

In patients surviving out-of-hospital cardiac arrest, targeted temperature management (TTM), previously known as mild therapeutic hypothermia, has been reported to significantly improve long-term neurological outcome and may prove to be one of the most important clinical advancements in the resuscitation science [8].

Clinical benefit of therapeutic hypothermia in patients with post-cardiac arrest syndrome (PCAS) has been demonstrated by two randomized control trials since 2002 [15, 16]. However, the term “therapeutic hypothermia” has been replaced with “targeted temperature management (TTM)” since 2011 after the meeting of five major professional physician societies [17]. Subsequently, a large multicenter study comparing TTM between 33 and 36 °C did not show the advantage of 33 °C above 36 °C [18]. Therefore, it is proposed that TTM treatment should be administered to OHCA patients with a shockable initial rhythm. The OHCA patients with ventricular fibrillation (VF) are the main indications for TTM. Therefore, it is necessary to establish a system to perform the TTM for resuscitated patients admitted to CICU. Doctors and nurses who work at CICU are required to have knowledge and skills on TTM.

Cardiac arrest, a sudden stop in effective blood flow, often happens outside the hospital. It is difficult for many patients who experience out-of-hospital cardiac arrests to survive. The most common cause of cardiac arrest is heart attack, and the most effective treatment for cardiac arrest is immediate cardiopulmonary resuscitation (CPR) and defibrillation by anyone who can do these procedures. The term, “Chain of Survival” is guidelines to help people survive cardiac arrest [19]. The five guidelines in the adult out-of-hospital Chain of Survival that are recommended by the American Heart Association (AHA) are:

1. Recognition of cardiac arrest and activation of the emergency response system
2. Early cardiopulmonary resuscitation with an emphasis on chest compressions
3. Rapid defibrillation
4. Basic and advanced emergency medical services
5. Advanced life support and post-cardiac arrest care

Recently, it is reported that the proportion of OHCA patients with a favorable neurological outcome improved significantly after the implementation of the fifth link [20]. TTM is included in the treatment for post-cardiac arrest syndrome (PCAS) which is on the fifth chain. TTM can be induced and maintained with basic means such as ice packs, fans, cold air blankets, and infusion of cold fluids or with costly advanced systems such as surface cooling pads or endovascular catheters [21]. Recently, a multicenter study comparing the effects of surface cooling and endovascular cooling was conducted [22]. Endovascular cooling appears to be more efficient in rapidly reaching and better controlling the targeted temperature with a decreased workload for nurses during the TTM period. However, endovascular cooling was not significantly superior to basic surface cooling in terms of favorable outcome. CICU medical staff needs to become proficient in using various devices for body temperature management.

## Management of cardiogenic shock in the CICU

Cardiogenic shock is a condition in which insufficient organ perfusion occurs due to decreased cardiac output [23]. Causes of cardiogenic shock include severe heart diseases such as AMI, fulminant myocarditis, and cardiomyopathy. This life-threatening emergency condition requires intensive monitoring with aggressive hemodynamic support. In order to survive patients with cardiogenic shock, resuscitation treatment must be performed before irreversible damage to important organs occurs.

The key to good outcome in patients with cardiogenic shock is a systematic approach, with rapid diagnosis and rapid start of pharmacological treatment to maintain blood pressure and cardiac output as well as treatment for the underlying disease. Pulmonary artery catheter is a useful method for evaluating the hemodynamics of shock patients. All shock patients require admission to general ICU or CICU. A multidisciplinary cardiogenic shock team is recommended to guide the rapid and efficient use of these available treatments [23]. All patients with cardiogenic shock require close hemodynamic monitoring, volume support to ensure adequate sufficient preload, and ventilatory support such as tracheal intubation and mechanical ventilation [24]. In mechanical circulatory support such as IABP, PCPS should be considered for patients with shock refractory to conventional medical therapy [23]. Cardiogenic shock is a clinical condition with high mortality rate. Further improvement of cardiovascular intensive care is expected to improve the life-saving rate of cardiogenic shock.

## Conclusions

Cardiovascular intensive care unit (CICU) is a hospital ward that specializes in the care of patients who have experienced ischemic heart disease as well as other severe heart disease. Furthermore, the patients in the CICU often have various complications such as respiratory failure and renal failure. Therefore, medical staffs who work at CICU are required to have the ability to practice systemic intensive care.

## Abbreviations

AHA: American Heart Association
AMI: Acute myocardial infarction
CAD: Coronary artery disease
CCU: Coronary care unit
CICU: Cardiovascular intensive care unit
CPR: Cardiopulmonary resuscitation
CVD: Cardiovascular disease
ECG: Electrocardiography
IABP: Intra-aortic balloon pump
ICU: Intensive care unit
OHCA: Out-of-hospital cardiac arrest
PCAS: Post-cardiac arrest syndrome
PCI: Percutaneous coronary intervention
PCPS: Percutaneous cardiopulmonary support system
SV: Stroke volume
TTM: Targeted temperature management
